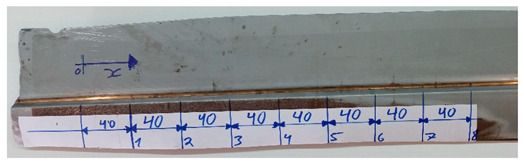# Multisensor Inspection of Laser-Brazed Joints in the Automotive Industry

**DOI:** 10.3390/s21217335

**Published:** 2021-11-04

**Authors:** Miguel A. Machado, Luís S. Rosado, Nuno M. Mendes, Rosa M. Miranda, Telmo G. Santos

**Affiliations:** 1 UNIDEMI, Department of Mechanical and Industrial Engineering, NOVA School of Science and Technology, NOVA University Lisbon, 2829-516 Caparica, Portugal; nam.mendes@fct.unl.pt (N.M.M.); rmmdm@fct.unl.pt (R.M.M.); telmo.santos@fct.unl.pt (T.G.S.); 2 Instituto de Telecomunicações, 1049-001 Lisbon, Portugal; luis.rosado@tecnico.ulisboa.pt; 3 Departamento de Engenharia Eletrotécnica e de Computadores, Instituto Superior Técnico, Universidade de Lisboa, 1049-001 Lisbon, Portugal

**Keywords:** non-destructive testing, eddy currents, potential drop method, laser brazing, automotive industry, numerical simulation, customized probes, four-point probe

## Abstract

Automobile laser brazing remains a complex process whose results are affected by several process variables that may result in nonacceptable welds. A multisensory customized inspection system is proposed, with two distinct non-destructive techniques: the potential drop method and eddy current testing. New probes were designed, simulated, produced, and experimentally validated in automobile’s laser-brazed weld beads with artificially introduced defects. The numerical simulations allowed the development of a new four-point probe configuration in a non-conventional orthogonal shape demonstrating a superior performance in both simulation and experimental validation. The dedicated inspection system allowed the detection of porosities, cracks, and lack of bonding defects, demonstrating the redundancy and complementarity these two techniques provide.

## 1. Introduction

Laser brazing offers diversified advantages for sheet metal joining of automobile assemblies. The comparison with fusion-based options shows lower thermal distortion, high-quality surfaces with no additional sealing required, easy automation and simplified clamping/fixtures, enhanced design freedom, increased productivity [1]. These advantages make laser brazing a standard and well-accepted manufacturing process, especially for joints fabricated in visible areas such as roof and tail gates [2].

Despite its wide acceptance in the industry, laser brazing remains a complex process whose results are affected by several process parameters and other factors, such as defects in the material’s microstructure, contaminations on the working piece, and modifications of the laser beam properties, which may result in nonacceptable welds [3]. Three main types of defects may occur: individual or clusters of pores; voids, discontinuities, lack of bonding and one-side wetting; surface irregularities such as scaly or wavy seams and irregularities with unfused filler material. These defects may affect both weld aesthetics and mechanical performance, potentially compromising performance under fatigue loading [3]. Therefore, the industry employs process monitoring and post-process inspection seeking for enhanced weld quality.

Different Non-Destructive Testing (NDT) has been applied for post-process inspection of laser-brazed joints. Optical approaches as the laser triangulation allowed to estimate the morphology and height of a joint while algorithms recovered the three-dimensional joint profile alongside detecting defects. This approach is limited to low-speed inspections and to the detection of surface defects that could otherwise be recognized with simple visual inspection [4]. Image processing of welded joint surfaces was used for defects’ detection and classification. As no depth information can be retrieved, it is impossible to recover the joint profile, and therefore, results are close to those of visual inspection with the benefits of automation [5]. Buried defects lack of detection and low speed of inspection are again the main drawbacks of this option. A combination of the two approaches was also tried [6], yet, the inability to detect relevant subsurface defects remains.

Electromagnetic NDT methods were also tried with interesting results regarding the detection of the several defect types. The induced Current Potential Drop (CPD) method was demonstrated to detect and estimate the depth of surface defects [7]. Eddy Current Testing (ECT) early demonstrated the ability to detect and classify defects [8], while the use of array probes, which allow enhanced throughput, was later proposed [9]. More recently, an innovative ECT inspection system was proposed for the detection of laser-brazed joints surface and sub-surface defects, operating autonomously and integrated with a robotized arm [10].

CPD methods rely on the analysis of the electrical current distribution in the part under inspection [11]. The method has two main variants, where either direct current (DCPD) [12] or alternate current (ACPD) [13] is applied within the part. Current is injected with two excitation electrodes/contacts while two sensing instances are used to read current distribution modifications, forming a so-called four-point probe. Application examples include the inspection of electronic cabling [14] and connectors [15], monitoring cracks under fatigue loading [16,17,18,19], the estimation of geometrical dimensions as thickness [20] and weld nugget [21], and the mapping of local conductivity [22] and mechanical stress [23]. Provided an available access to the part conductive surface, CPD methods are a simple, reliable, and inexpensive NDT option. Nevertheless, when probes need to move along the inspection surface, contacts may suffer excessive wearing, and variations on the contact electrical resistance appear as a signal perturbation.

ECT is an electromagnetic NDT method based on inducing and sensing electrical current, the Eddy Currents (EC), on the superficial layers of electrically conductive parts [24]. On its conventional variant, the impedance of a sensor coil is measured while being energized with an alternating current. An alternate magnetic field is generated, which is responsible for inducing EC on the part. Once an interaction between the EC and the inspected part occurs and defective conditions appear, its distribution and consequently the magnetic field distribution are modified. This modification is ultimately sensed as a change on the coil impedance. ECT has been broadly applied for contactless measurements on electrically conducting parts including metals [25,26], composites [27], and even some fibers [28,29]. The method is well suited for cracks detection [30,31] as well as to perform coating thickness [32] and conductivity measurements [33] which are related to bulk grain size and porosity.

This paper discusses the application of both DCPD and ECT methods for the inspection of laser-brazed joints fabricated in automobile assemblies. An innovative inspection system combining the two methods provided promising results by profiting from the methods redundancy and complementarity.

## 2. Materials and Methods

**Figure 1:**
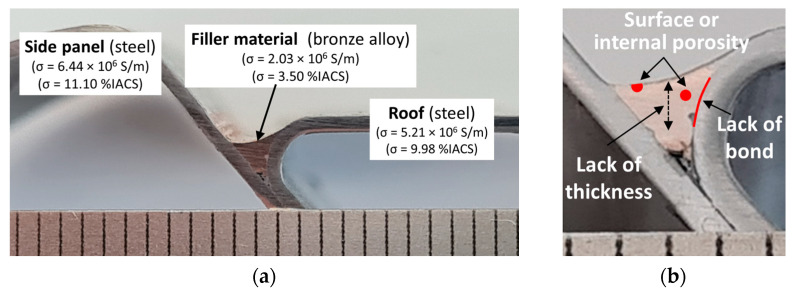
Laser-brazed weld profile section between the side panel and the roof steel sheets (scale in mm). (**a**) General overview; (**b**) detail of possible defects morphology and location.

The laser-brazed joint considered across this study was located on a car roof and joined two 0.7 mm-thick different steels panels: the roof panel and the side panel, as depicted in Figure 1a. The filler material was a silicon bronze alloy commonly used in these joints [1]. The electrical conductivity of the materials is crucial for the design of dedicated inspection probes, so measurements were carried out. The roof panel had an electrical conductivity of 5.21 × 10^6^ S/m, the side panel of 6.44 × 10^6^ S/m, and the filler material of 2.03 × 10^6^ S/m [10]. Figure 1b schematically illustrates the possible defects that may occur in such joints. Because of the difference between the panels’ materials, the non-flat geometry and the limited accessible surface, the joint presents a very demanding inspection scenario.

The developed inline inspection system comprised two distinct NDT techniques bringing redundancy and complementarity and increasing the results’ reliability across all defects’ morphologies.

**Figure 2:**
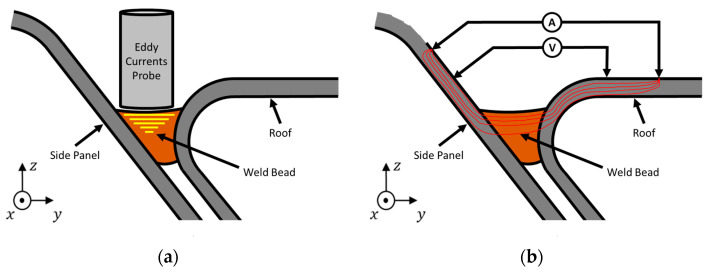
Inspection methods envisaged. (**a**) Eddy currents testing (**b**) potential drop measurement.

One of the techniques employed an eddy current probe customized to fit the weld bead and aiming at the detection of porosity defects or surface and sub-surface cracks in the brazed bead (Figure 2a). The other technique consisted of the direct current potential drop (DCPD) method which makes use of a four-point probe. This technique targets the detection of surface and internal porosities as well as the lack of bonding between the steel sheets and the filler material. The DCPD method probes are usually built with a set of four very small needles disposed in a straight line [22], although a rectangular configuration can also be used [34]. The electric current is imposed through the outer needles, and the potential drop is measured between the two inner needles. For the same imposed current, the greater the potential drop, the greater the resistivity of the material, for which closed-form analytical solutions are well known [22]. However, the inspection can be lengthy because conventional probes cannot be dragged along the weld. Instead, the probes must be recessed while travelling between the tested positions, and the needles are very sharp and may damage the weld surface or wear out. Commercially available probes are designed for planar surfaces, which means that all the needles have the same length. The use of DCPD in this application required a customized probe to overcome these limitations.

On a first approach, we was used a DCPD probe with measurement points as presented in Figure 2b. All four pins were in the same YOZ plane, two on the side panel, and two on the roof panel to assure the entire weld was crossed by the electric current, including the bimetallic interfaces bronze/steel. The current was forced to travel through the filler material and if a defect appeared, the current path would change causing a potential drop. Instead of needles, spring-loaded connectors were used to allow the probe to be dragged along the weld bead performing a continuous reading. Numerical simulations were carried out to understand the applicability of the proposed configuration.

## 3. Results

### 3.1. Numerical Simulation

**Figure 3:**
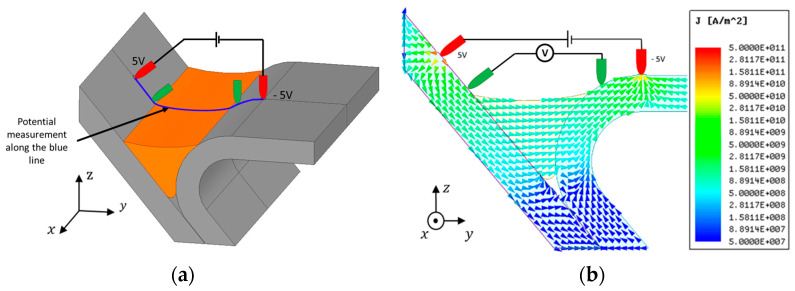
Four-point probe numerical simulation: (**a**) pins positioning towards the profile; (**b**) current flow through the weld.

Numeric simulations were performed aiming to understand electric and magnetic phenomena involved in the probe operation with this complex geometry and materials. A CAD model was designed (Figure 3a) from a profile section of a digitalized weld bead (from Figure 1) to be as similar as possible to reality. The measured material properties were used within the model. The numerical simulation software ANSYS Electronics was used, allowing to calculate an approximate numerical solution of Maxwell’s equations in their full formulation, Finite Integration Technique (FIT). Two million tetrahedral elements were used to perform these simulations. An electric potential difference of 10 V was imposed between the two outer pins.

The potential drop measurement method was simulated to understand the best four-point configuration. The first approach consisted in placing the four points in the same vertical plane according to Figure 3a. The voltage was imposed between the outer pins represented in red in Figure 3a, and the potential drop was measured between the two inner pins represented in green. Figure 3b allows to observe the currents path through the material when there was no defect present.

**Figure 4:**
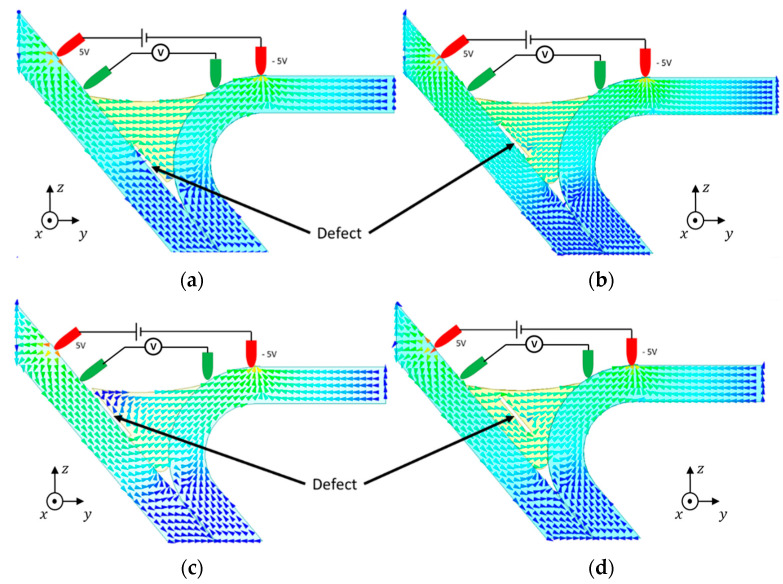
Current density vector field behavior in a weld with different defect locations.

**Figure 5:**
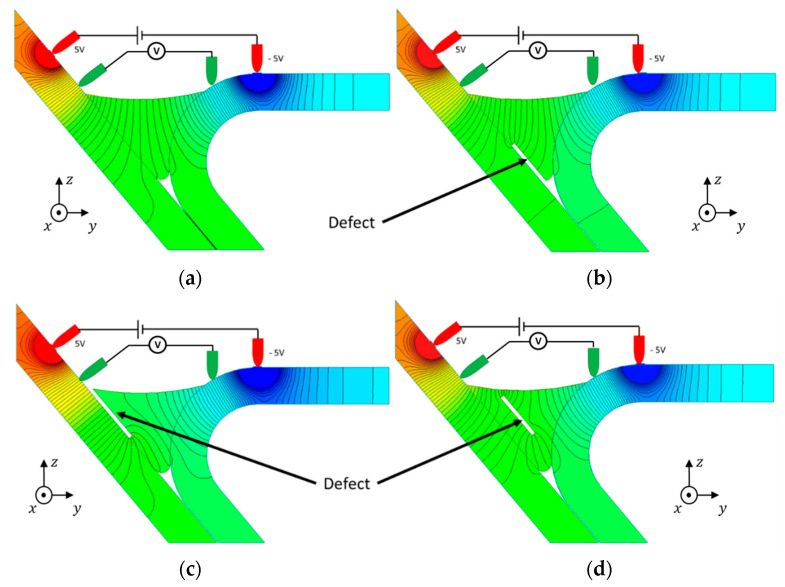
Electric equipotential lines along the current flow direction for different defect locations.

Figure 4 shows the results obtained with different 0.2 mm-thick defect locations. The current flow was deviated by the defects. However, since the potential drop was our indicator, Figure 5 depicts the potential along the current flow. These results acknowledge that there were changes in the current flow and potential throughout the weld in the presence of defects. The current density vector field is greatly affected by surface and sub-surface defects, while defects away from the inspection surface do not affect the current density vector field as much.

**Figure 6:**
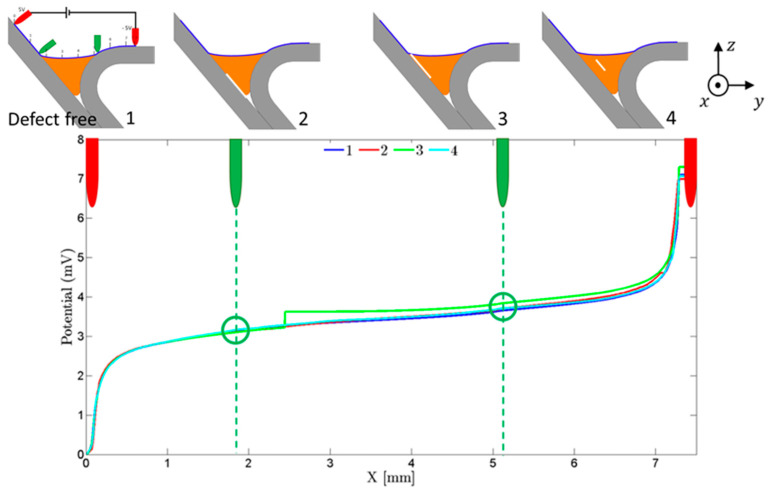
Potential drop along the transversal line that contained all the pins considering four different defects location. The maximum potential drop occurred in the position X ≈ 2.5 mm for the defect type N°3, but the potential drop measured (green pin position) was quite low. (Legend: 1—No defect; 2—Internal lack of bonding; 3—Surface lack of bonding; 4—Internal weld bead defect).

Despite the importance of observing the behavior of the electric currents flow in the material, in practice only two inner points were available for measurements. In Figure 6, a blue line was designed between the two outer points (where current was imposed) along the surface, and a plot was built with the potential along that line. Thus, a careful analysis was conducted to understand if the potential drop between the selected points (inner pins) was significant or if a different location was preferred. These two pins must necessarily be outside the weld bead to ensure that any defect in the weld is between them (including lack of bonding). However, as shown in Figure 6, the potential drop between the green pins was not noticeable either with or without defects and was much lower with different defect locations. For example, in the case number, 3 a potential drop of 0.5 mV occurred somewhere between the sensing pins, but the measured potential difference was much lower, being less than 0.1 mV. For this reason, other ways of solving this problem were sought.

**Figure 7:**
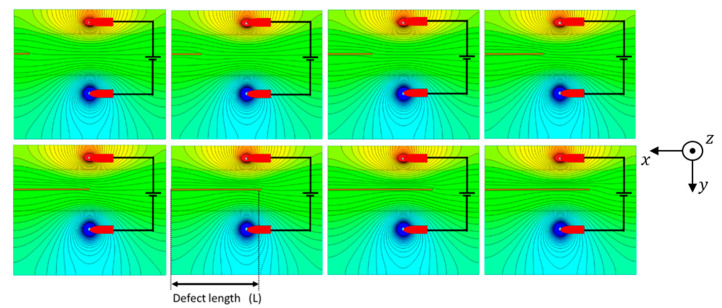
Electric equipotential lines from the top of the weld bead for an increasing defect in the X direction.

**Figure 8:**
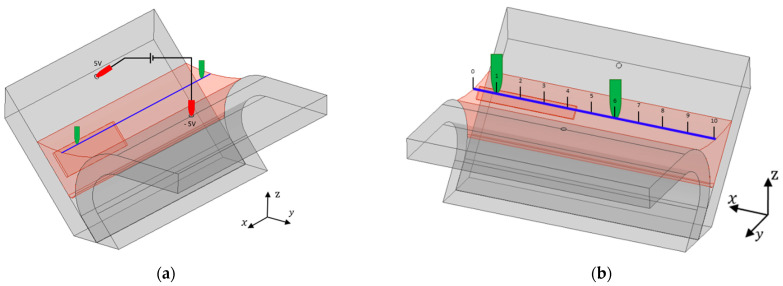
New pin distribution proposed with an orthogonal configuration. (**a**) Concept solution with the orthogonal configuration; (**b**) one possible location for the two sensing pins.

A new approach consisted in evaluating the equipotential lines distribution on the entire surface of the weld and base materials, searching for the positions that maximized the potential drop caused by the defects. Figure 7 depicts the equipotential lines on the joint surface with a defect length increasing along the X direction. These results allow to conclude that aligned pins could not be the best solution to obtain the highest potential drop. In fact, Figure 7 suggests that an orthogonal positioning of the four pins, maintaining the outer pins in their position and placing the sensitive ones over the joint apart from each other, could be a better solution to obtain a higher potential drop (Figure 8a).

**Figure 9:**
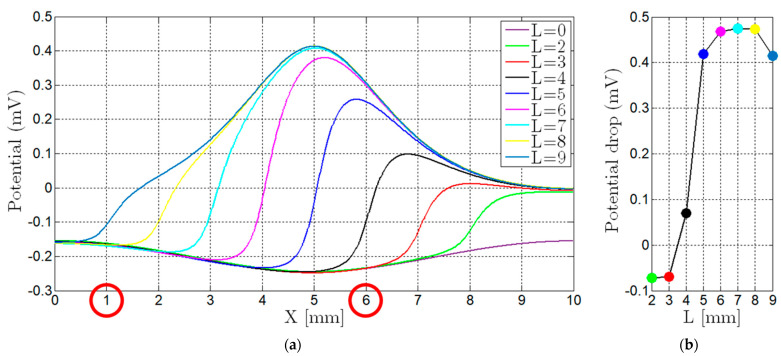
Numeric simulation results. (**a**) Potential distribution along the blue longitudinal line from Figure 8 with the increase of the defect length L from Figure 7. The positions 1 and 6 correspond to the overall maximum potential drop for the different defect’s lengths; (**b**) potential drop between the two chosen pin locations for different defect lengths L.

Several simulations were performed, and the potential along a longitudinal line in the middle of the weld bead (blue line in Figure 8b) was evaluated. In Figure 9a, each line represents the potential variation along the weld bead for each defect length (L) in the X direction (Figure 7). From these curves, two locations were selected in which the overall potential drop was higher, resulting in positioning the sensitive pins at 1 and 6 mm in the X axis, as in Figure 8b.

The potential drop between these locations was computed and is represented in Figure 9b. Each dot represents the potential difference between the two marked locations in Figure 9a for each defect length L, displayed with the same color as the curve from which it was calculated. This orthogonal pattern for pins location resulted in a potential drop between the sensing pins of around 0.5 mV, which corresponded to a 500% improvement compared with the conventional straight line probe configuration.

### 3.2. Four-Point Probe for Potential Drop Measurement

**Figure 10:**
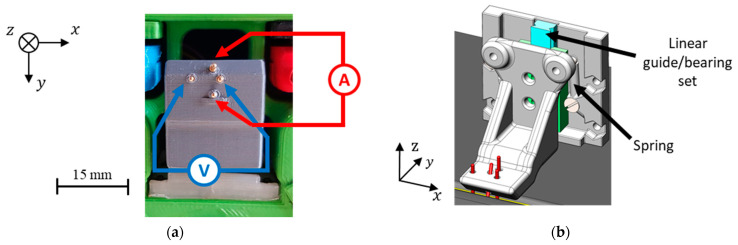
Four-point probe based on the numerical simulation-based preferred arrangement of the pins (orthogonal pattern). (**a**) Produced probe; (**b**) CAD model.

Based on the numerical simulation results, a new four-point probe was designed and manufactured with the pins arrangement in an orthogonal disposition, as depicted in Figure 10a. The needles consisted of spring-loaded connectors with a rounded end. The springs ensured the sustained contact between the pins and the weld bead. The spring-loaded connectors were assembled in a polymeric 3D printed part which held them in the desired position. This 3D printed part was connected to the scanning device through a small linear bearing, spring-loaded as well, which allowed the macro-positioning of the four-point probe over the weld bead, as shown in Figure 10b.

### 3.3. Eddy Current Probes

Tailored-made eddy current probes were designed to fit the chassis of the prototype inspection device. Bobbin coils with a reduced diameter were produced specifically to fit the weld bead.

**Figure 11:**
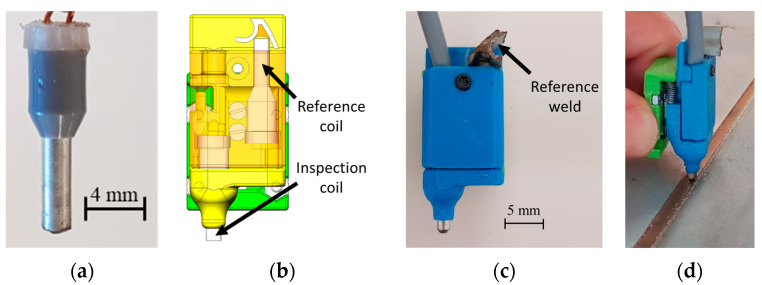
Tailored EC probes. (**a**) Encapsulated EC individual bobbin; (**b**) EC probe CAD representation; (**c**) probe with two bobbins which operate in bridge differential mode; (**d**) device over the welded joint coupled with a spring-loaded linear bearing for a constant lift-off.

The joint materials and geometry were necessarily a constraint to the EC probes design. The probes operation frequency range was firstly calculated bearing in mind the required penetration depth. Consequently, low-frequency probes for sub-surface defects and higher-frequency probes for surface defects were designed, numerically simulated, and produced. Different design options such as number of windings, wire material and diameter, core material and dimensions, shielding material and dimensions were specified, since they highly influence the probe frequency response. The designed probes are composed of two cylindrical helicoidal bobbin coils (Figure 11a) operating in a bridge differential mode. One of the bobbins moves over the weld bead, while the other has the opposite orientation (Figure 11b), with a reference weld in good conditions, as depicted in Figure 11c. A 3D-printed probe holder was designed and produced to hold two bobbin coils together and was attached to the scanning device by a linear bearing and a spring which ensured a constant lift-off, as shown in Figure 11d. Operating in bridge differential allowed a greater sensibility, since the inspected weld was compared with a good one.

### 3.4. Scanning Device

**Figure 12:**
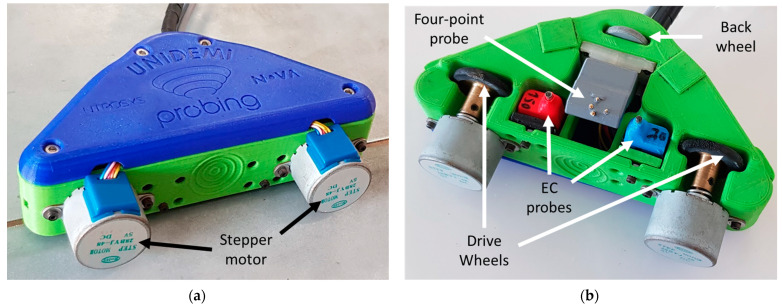
Scanning device comprising both customized eddy currents and four-point probe. (**a**) Top view of the device over a weld specimen; (**b**) bottom view.

In order to automatize the inspection process, a scanning device was designed, and 3D-printed by Fused Deposition Modeling (FDM) in polylactic acid (PLA), as shown in Figure 12. The scanning device held each probe individually through linear bearings and springs, as mentioned before. The scanning device is composed of three wheels, two drive wheels which move the device while guided by the welded joint profile, and a third wheel remaining over the roof panel. Two small magnets were also placed inside the scanning device chassis to ensure a good coupling of the scanning device to the welded panels. The two drive wheels were activated by stepper motors which were responsible for controlling the scanning device movement. Additionally, the scanning device was designed for handheld manual operation or to be attached to an industrial robotic arm.

### 3.5. Experimental Results

#### 3.5.1. Sample Description

Experimental tests were accomplished in laboratory environment using a specimen with artificially made defects. Drilled holes and electrical discharge machining (EDM) defects were produced, as indicated in Table 1. Different defects, with different locations, dimensions, and morphologies, were created to mimic real defects.

#### 3.5.2. Eddy Currents and Analysis

**Figure 13:**
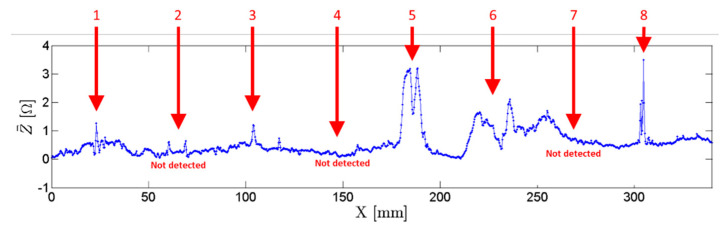
Customized EC probe output signal operating at a frequency of 750 kHz. Defects 1 to 8, as presented in Table 1.

For EC testing, several operating frequencies were used ranging from 150 kHz to 2 MHz, and the 750 kHz frequency achieved the best results. Figure 13 presents an eddy current test at the frequency of 750 kHz, performed with the inspection system illustrated in Figure 12, operating with a Nortec 600D commercial EC instrument f (Olympus, Tokyo, Japan). Defects 2, 4, and 7 did not create a visible signal indication, since they were too close to the interface and therefore too far away from the EC probe. A noticeable signal output was measured for defect 5, which was at the weld bead/panel interface. For this situation, the probe scanned near this interface. It would be tempting to design a probe whose outside diameter is equal to the space between interfaces; however, that could result in the probe becoming stuck or not fitting inside the available space if the produced weld profile slightly decreases from its nominal. The remaining defects were detected even by the raw results to which any sort of filtering or post-processing had yet to be applied.

One of the main advantages of the EC technique for this specific application relies on its lack of response to the presence of non-conductive materials. Therefore, EC signals relate only to the weld bead and base materials. In image-based inspection systems like simple visual inspection [4], image processing [5], laser vision [35], or near-infrared signals [36], any dust or dirt over the bead may be identified as false positive. These approaches also miss the ability of assessing conductivity variations potentially revealing excessive internal porosity and other metallurgical deviations.

**Figure 14:**
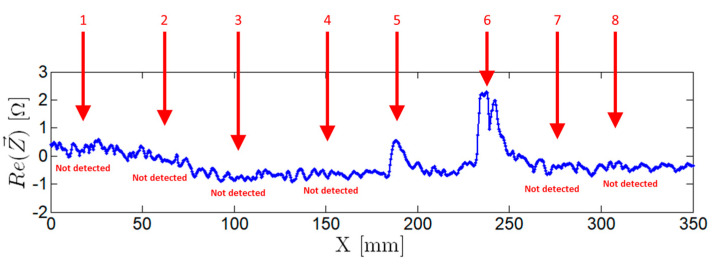
Commercial EC probe output signal operating at a frequency of 750 kHz. Defects 1 to 8 as presented in Table 1.

A commercial pencil probe, operating in the same conditions, was also tested in the same sample, and the results can be seen in Figure 14. Only two defects (5 and 6) were detected with this probe, evidencing improved reliability of the developed EC probes.

#### 3.5.3. Potential Drop Measurement

**Figure 15:**
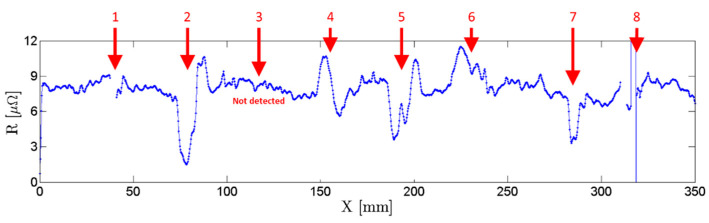
Developed orthogonal-shape four-point probe output signal. Defects 1 to 8 as presented in Table 1.

**Figure 16:**
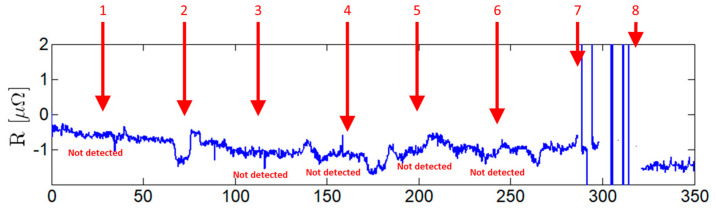
Straight line four-point probe output signal. Defects 1 to 8 as presented in Table 1.

Potential drop measurements were performed using the dedicated four-point probe on the same sample and simultaneously with EC testing shown in Figure 13. A current of 1 A DC was imposed on the outer pins in both directions by a source meter unit Keithley 2450 (Tektronics, Beaverton, OR, USA). Figure 15 depicts the output of the four-point probe. This technique allowed the detection of defects near the interfaces, the locations where the EC probes did not perform. A straight line four-point probe was also developed and produced for an experimental comparison. As seen in Figure 16, the straight line four-point probe performance was lower compared to that of the orthogonally shaped one, as suggested by numeric simulations.

The four-point technique can be a great advantage when lack of fusion defects are present. Lack of fusion defects are located at the interface between the filler material and the sheet metal and not always open to the surface, becoming hardly detected by surface-scanning techniques such as visual inspection [4,5,35,36]. In addition, these techniques’ output consists of multi-dimensional data for each measured point that must be stored and potentially processed offline. On the other hand, both EC and DCPD techniques produce a one-dimensional output much more appealing for storage and online real-time processing.

## 4. Discussion and Conclusions

A multisensor inspection system for the inspection of laser-brazed joints was developed. By featuring two distinct non-destructive techniques, it allowed redundancy and complementarity on the inspection process. The proposed inspection device combined two complementary electromagnetic techniques: eddy currents and potential drop measuring.

Both techniques were not easily applicable to the inspection conditions, since there are no commercially available probes that could be correctly applied to the weld bead complex geometry. Dedicated probes were designed, simulated, produced, and experimentally validated.

Numerical simulations were crucial for the design of the four-point probe. The linear conventional pins alignment was numerically simulated and was considered unfit for this specific application. Different pin alignments were simulated, and a non-obvious orthogonal pin arrangement was found to allow substantial sensitivity enhancements of up to 500%.

A scanning device was designed and produced with polymer FDM additive manufacturing and allowed the eddy currents and four-point probes movement along the joint. The system was experimentally validated on a sample with several reference artificial defects. The redundancy and complementarity of the inspection device was verified, since some defects were detected by both techniques, some only by the eddy currents probe, and some others only by the four-point probe. Defects in the middle of the joint, like cracks and porosities, were more easily detected by eddy currents while defects closer to the interface, simulating lack of bonding, were more appropriately inspected with potential drop measurement.

## Figures and Tables

**Table 1:** Artificial defects produced in the sample to be inspected (scale in mm).

N°	Picture	N°	Picture
1	Surface welded defect 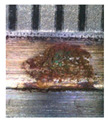	5	Interface defect between steel and weld 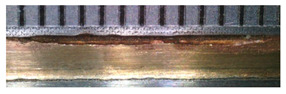
2	Surface welded defect 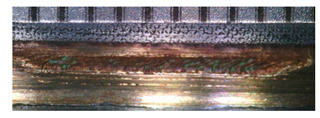	6	Interface defect between steel and weld 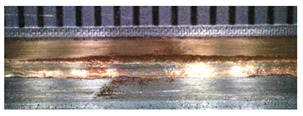
3	Interface defect between steel and weld 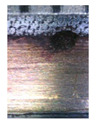	7	Interface defect between steel and weld 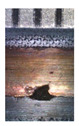
4	Interface defect between steel and weld 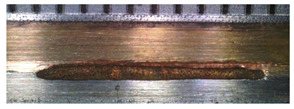	8	Surface welded defect 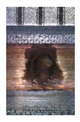
Test piece